# Determination of Chemical Composition and Investigation of Biological Activities of *Ocimum basilicum* L.

**DOI:** 10.3390/molecules28020614

**Published:** 2023-01-06

**Authors:** Ahmed Qasem, Hamza Assaggaf, Hanae Naceiri Mrabti, Faisal Minshawi, Bodour S. Rajab, Ammar A. Attar, Reema A. Alyamani, Munerah Hamed, Nidal Naceiri Mrabti, Aicha El Baaboua, Nasreddine El Omari, Mohammed Merae Alshahrani, Ahmed Abdullah Al Awadh, Ryan Adnan Sheikh, Long Chiau Ming, Khang Wen Goh, Abdelhakim Bouyahya

**Affiliations:** 1 Department of Laboratory Medicine, Faculty of Applied Medical Sciences, Umm Al-Qura University, Makkah 21955, Saudi Arabia; 2 Laboratoires TBC, Faculty of Pharmaceutical and Biological Sciences, B.P. 8359006 Lille, France; 3 Faculty of Applied Medical Sciences, Clinical Nutrition Department, Umm Al-Qura University, Makkah 24381, Saudi Arabia; 4 Department of Pathology, Faculty of Medicine, Umm Al-Qura University, Makkah 21955, Saudi Arabia; 5 Engineering Materials, Modeling and Environmental Laboratory, Faculty of Sciences Dhar El Mehraz, Sidi Mohammed Ben Abdellah University, Fez 30000, Morocco; 6 Biology and Health Laboratory, Department of Biology, Faculty of Science, Abdelmalek-Essaadi University, Tetouan 93000, Morocco; 7 Laboratory of Histology, Embryology and Cytogenetic, Faculty of Medicine and Pharmacy, Mohammed V University in Rabat, Rabat 10100, Morocco; 8 Department of Clinical Laboratory Sciences, Faculty of Applied Medical Sciences, Najran University, P.O. Box 1988, Najran 61441, Saudi Arabia; 9 Biochemistry Department, Faculty of Science, King Abdulaziz University, Jeddah 21589, Saudi Arabia; 10 School of Medical and Life Sciences, Sunway University, Bandar Sunway 47500, Malaysia; 11 Faculty of Data Science and Information Technology, INTI International University, Nilai 71800, Malaysia; 12 Laboratory of Human Pathologies Biology, Department of Biology, Faculty of Sciences, Mohammed V University in Rabat, Rabat 10100, Morocco

**Keywords:** cosmetology, aromatherapy, essential oils, biological properties, biomedicine, basil

## Abstract

This study aimed to determine the chemical composition of the essential oils (EOs) of *Ocimum basilicum* L., as well as to evaluate the antibacterial, antidiabetic, dermatoprotective, and anti-inflammatory properties, and the EOs and aqueous extracts of *O. basilicum*. The antibacterial activity was evaluated against bacterial strains, Gram-positive and Gram-negative, using the well diffusion and microdilution methods, whereas the antidiabetic activity was assessed in vitro using two enzymes involved in carbohydrate digestion, α-amylase and α-glucosidase. On the other hand, the dermatoprotective and anti-inflammatory activities were studied by testing tyrosinase and lipoxygenase inhibition activity, respectively. The results showed that the chemical composition of *O. basilicum* EO (OBEO) is dominated by methyl chavicol (86%) and *trans*-anethol (8%). OBEO exhibited significant antibacterial effects against Gram-negative and Gram-positive strains, demonstrated by considerable diameters of the inhibition zones and lower MIC and MBC values. In addition, OBEO exhibited significant inhibition of α-amylase (IC_50_ = 50.51 ± 0.32 μg/mL) and α-glucosidase (IC_50_ = 39.84 ± 1.2 μg/mL). Concerning the anti-inflammatory activity, OBEO significantly inhibited lipoxygenase activity (IC_50_ = 18.28 ± 0.03 μg/mL) compared to the aqueous extract (IC_50_ = 24.8 ± 0.01 μg/mL). Moreover, tyrosinase was considerably inhibited by OBEO (IC_50_ = 68.58 ± 0.03 μg/mL) compared to the aqueous extract (IC_50_ = 118.37 ± 0.05 μg/mL). The toxicological investigations revealed the safety of *O. basilicum* in acute and chronic toxicity. The finding of in silico analysis showed that methyl chavicol and *trans*-anethole (main compounds of OBEO) validate the pharmacokinetics of these compounds and decipher some antibacterial targets.

## 1. Introduction

Medicinal plants are increasingly used as sources of bioactive compounds required for drug development [1]. The World Health Organization (WHO) estimates that approximately 80% of the population in developing countries depend on medicinal plants and traditional medicine for their primary health care [2]. In this line, the study of the biological activities of natural molecules has often been addressed through three main approaches, namely, selection based on chemotaxonomy, selection of random natural sources, and selection based on ethnomedicine [3]. However, ethnobotanical studies are the most critical parameter in the selection of plants to be tested for their biological properties [4,5,6,7,8,9]. The floristic diversity of Morocco is undoubtedly due to its biogeographical position [10]. In addition, several ethnobotanical studies have demonstrated the history and expertise of this country in the use of medicinal plants [11,12,13]. 

The genus Ocimum, belonging to the Lamiaceae family, includes about 150 species [14], the majority of which are used in the treatment of multiple types of ailments from antiquity, in particular the species *Ocimum basilicum* L. [15], also called sweet basil, which is a universally cultivated, perennial herbaceous plant [16]. This plant is used as an ornamental herb, a culinary herb, and a kitchen herb [17]. Its essential oils (EOs) are used as food flavorings, commercial fragrances, and food preservatives [18,19,20]. It has been linked to many fields such as pharmacology, food, agriculture, and cosmetics.

Indeed, *O. basilicum* (basil) is one of the most important medicinal plants in Morocco. In fact, it is a vital oil-bearing plant that can be grown under various environmental conditions in a wide range of terrains.

There are certain diseases that can affect basil, the most important of which are leaf spot, Botrytis, and Fusarium. In fact, previous studies have reported that basil is sensitive to fresh and dry matter deficiency, drought stress, and certain chemical constituents (carbohydrate, proline, and protein) [21,22].

Traditionally, basil (taken as an herbal tea) has been used in Morocco to treat numerous diseases and disorders, including diabetes, infectious diseases, sinusitis, tachycardia, hemorrhoids, and inflammation [23]. In other countries, this plant has been used against earache problems, kidney disorders, diarrhea, coughs, colds, headaches, insect bites, nerve pains, cardiovascular diseases, sinusitis, menstrual cramps, migraines, fevers, and anxiety [24,25,26]. Several articles have investigated the biological properties of *O. basilicum*, including antioxidant [27,28,29], antimicrobial [30,31,32,33,34], anticancer [35,36], immunomodulatory [37], insecticide [38], anti-thrombotic [39] antiplatelet [40], anti-inflammatory [41], anti-hyperlipidemic [40], and anticonvulsant [38,42] effects. However, other pharmacological properties such as dermatoprotective effects have not been investigated.

Recent investigations have shown that various factors such as growth stage, harvest season, ecological conditions, and the part of the plant used are involved in the growth of medicinal plants and their content of bioactive compounds [43,44,45,46].

In addition, the biological and pharmacological properties of medicinal plants are related to different bioactive compounds synthetized as secondary metabolites by these plants. The diversity of these chemical groups justifies a medicinal plant’s pharmacological potential. Indeed, various secondary metabolites have been identified from basil, such as terpenoids (including methyl chavicol, geraniol, linalool, 1,8–cineole, methyl eugenol, p-allylanisole, neryl acetate, and trans–α–bergamotene), flavonoids, phenols, anthocyanins, steroids, and tannins [47,48]. The traditional uses of basil and its richness in bioactive compounds encourage researchers to study the biological activities of this plant. Accordingly, this study focuses on investigating the chemical composition of *O. basilicum*, as well as the antimicrobial, antidiabetic, dermatoprotective, and anti-inflammatory activities of its EO and aqueous extract.

## 2. Material and Methods

### 2.1. Plant Collection and Extraction

Aerial parts of *O. basilicum* were collected in Sahel Boutaher (34°29′52.3″ N 4°47′11.9″ W) in the Taounate region, northwest Morocco. The plants were identified and confirmed by botanists from the Botany Department of the Scientific Institute of Rabat, Morocco. Voucher specimens of each plant were deposited in the herbarium under the voucher specimen code RAB 10532. EO extraction was carried out by hydro-distillation in a Clevenger-type apparatus (VWR, Radnor, PA, USA). A total of 100 g of the dry matter of the aerial part was placed in water and boiled for 3 h. The oil was recovered and then stored at a temperature of 4 °C [46].

### 2.2. GC-MS Analysis of Essential Oils

As previously described, the chemical components of OBEO were determined using gas chromatography/mass spectrometry (GC/MS) analysis [49]. A Hewlett-Packard (HP6890) GC instrument coupled to an HP5973 MS and equipped with a 5% phenylmethyl silicone HP-5MS capillary column (30 m × 0.25 mm × film thickness 0.25 μm) was used in GC analysis. The used column temperature increased from 50 °C, for 5 min, to 200 °C with a rate of 4 °C/min. Helium with a 1.5 mL/min flow rate and split mode (flow: 112 mL/min, ratio: 1/74.7) was used as carrier gas. The hold time was 48 min, while the injector and detector temperature was 250 °C.

The instrument was equipped with a computer system type ″HP ChemStation″, managing the operation of the device and allowing to follow the evolution of the chromatographic analyses. Diluted samples (1/20 in methanol) of 1 μL were injected manually. Additionally, 70 eV ionization voltage, 230 °C ion source temperature, and 35–450 (m/z) scanning range were the MS operating conditions. Finally, quantification of the different compounds was based on the percent area of each peak of the sample compounds and was confirmed by reference to their MS identities (Library of NIST/EPA/NIH MASS SPECTRAL LIBRARY Version 2.0, build 1 July 2002).

### 2.3. Evaluation of Antimicrobial Activity 

#### 2.3.1. Microorganisms Tested

The antimicrobial activity of *O. basilicum* EO and aqueous extract was evaluated against six bacterial strains representing Gram-positive and Gram-negative bacteria, namely *Escherichia coli* ATCC 25922, *Proteus mirabilis* ATCC 25933, *Salmonella enterica* Typhimurium ATCC 700408, *Bacillus subtilis* ATCC 6633, *Staphylococcus aureus* ATCC 29213, and *Listeria monocytogenes* ATCC 13932, one clinical yeast (*Candida albicans*), and two clinical fungi (*Trichophyton rubrum* and *Aspergillus niger*).

#### 2.3.2. Inoculum Preparation

The bacterial strains were revived by inoculation in Mueller–Hinton Agar (MHA) followed by incubation at 37 °C for 24 h. A bacterial suspension (0.5 McFarland) was prepared in sterile physiologic water (0.9% NaCl) from the obtained culture. The yeast strain was inoculated in Sabouraud’s agar (SA) and incubated at 25 °C for 48 h, then a yeast suspension (0.5 McFarland) was prepared in sterile physiological water. The Fungi strains were sub-cultured in SA and incubated at 25 °C for 5 days. Afterward, the culture was washed with 2 mL of sterile physiological water. The suspension was transferred to a sterile assay tube, where the heavy particles were allowed to settle for 5 min. The upper homogeneous suspensions were transferred to a new sterile tube and adjusted microscopically to about 10^4^ CFU/mL. The inoculum of bacteria, yeasts, and fungi was used to evaluate the antimicrobial activity of *O. basilicum* EO and aqueous extract.

#### 2.3.3. Disc Diffusion Assay

The antimicrobial activity of *O. basilicum* EO and aqueous extract against the tested microorganisms was investigated using the disc diffusion method according to the previously published protocols [50,51]. Briefly, the culture suspension was inoculated by swab on optimal culture media (MHA for bacteria and SA for yeasts and fungi). Afterward, six mm diameter sterile paper discs soaked in 10 µL of EO (mixed with 5% dimethyl sulfoxide (DMSO)) or aqueous extract were deposited on each plate. Chloramphenicol (30 µg) was used as a positive control for bacteria, and nystatin (100 I.U.) was used as a positive control for yeasts and fungi, while DMSO (10 µL; 5%) as a negative control. Incubation was then conducted at 37 °C for 24 h, 25 °C for 48 h, and 25 °C for 72 h for bacteria, yeasts, and fungi, respectively. After incubation, the diameters of the inhibition zones were measured in millimeters, and the results were expressed as the mean ± standard deviation (SD) of three replicates.

#### 2.3.4. Determination of Minimum Inhibitory Concentration

The minimum inhibitory concentration (MIC) is defined as the lowest concentration of EO or extract that can inhibit the growth of microorganisms. MIC determination for bacteria and yeasts was performed according to the protocol described previously [50], with some modifications. Mueller–Hinton broth (MHB) (Biokar, Beauvais, France) was used for bacteria, whereas Sabouraud broth (Biokar, Beauvais, France) was employed for yeast. The incubation was carried out at 37 °C for 24 h for the bacteria and at 25 °C for 48 h for the yeasts. However, MIC determination for fungi was carried out by the gradient plate method according to the protocol previously described [51]. Chloramphenicol was used as a positive control for bacteria, while nystatin was used for yeasts and fungi.

#### 2.3.5. Determination of Minimum Bactericidal Concentration

Minimum bactericidal concentration (MBC) corresponds to the lowest EO or extract concentration that can kill the microorganism. The same microdilution experiment used in determining the MIC was used. After the incubation, 10 μL of each tube showing no visible growth was sub-cultured on tryptone soy agar (TSA) (Biokar, Beauvais, France) and incubated at 37 °C for 24 h. Then, the lowest concentration that showed no growth on the culture medium was considered as MBC [52].

### 2.4. Antioxidant Activity 

The antioxidant activity of *O. basilicum* EO and aqueous extract was evaluated using three complementary methods, namely 2,2- diphenyl-1-picrylhydrazyl (DPPH), ferric reducing antioxidant power (FRAP), and 2,2-azinobis (3-ethyl-benzothiazoline-6-sulfonic acid (ABTS) assays, according to previously published procedures [53,54,55]. The results are expressed as the concentration of EOs providing 50% inhibition, inhibitory concentration 50% (IC_50_), and calculated by plotting the inhibition degrees as a function of EO concentrations. Trolox and ascorbic acid were used as positive controls. Assays were performed in triplicate, and IC_50_ values are presented as means ± SD.

### 2.5. In Vitro Anti-Diabetic Assay

The anti-diabetic effect of *O. basilicum* EO and aqueous extract was determined by testing their ability to inhibit the enzymatic activity of α-amylase and α-glucosidase, according to the method outlined by Mrabti et al. [56], and the lipase inhibitory effect was evaluated according to the method described by Hu et al. [57]. For the α-amylase test, a volume of 250 μL of EO and 250 μL of 0.02 M sodium phosphate buffer (pH = 6.9) containing α-amylase at 240 U/mL were incubated for 20 min at 37 °C. Then, 250 μL of a 1% starch solution prepared in 0.02 M sodium phosphate buffer (Ph = 6.9) was added, followed by incubation for 15 min at 37 °C. Then, 1 mL of DNS was added, and the mixture was incubated in a boiling water bath for 10 min. The reaction mixture was diluted by adding 2 mL of distilled water and the absorbance was measured at 540 nm with a spectrophotometer. In this rest, acarbose was used as a positive control. For the α-glucosidase test, 200 μL of EO and 100 μL of 0.1 M sodium phosphate buffer (SPB) (pH = 6.7) containing the α-glucosidase enzyme solution (0.1 U/mL) were incubated at 37 °C for 10 min. After preincubation, 200 μL of 1 mM pNPG solution in 0.1 M SPB (pH = 6.7) was added. After incubation at 37 °C for 30 min, 1 mL of 0.1 M of Na_2_CO_3_ was added, and the absorbance was recorded at 405 nm. The inhibitory effects of these two digestive enzymes were expressed as percentage inhibition and the IC_50_ values were determined. 

### 2.6. 5-Lipoxygenase (5-LOX) Inhibition Assay

The lipoxygenase inhibitory activity of *O. basilicum* EO and aqueous extract was investigated using the method adopted by Andarade et al. [58]. Briefly, a pre-incubation of 20 µL of EO or extract with 20 µL of 5-LOX (Glycine max) at 100 U/mL with 200 µL of phosphate buffer (0.1 M, pH 9) was performed for 5 min at room temperature. Then, 20 µL of linolenic acid (4.18 mM in ethanol) was added to start the reaction, followed by 3 min at 234 nm [58]. Results are presented as the mean ± SEM of three independent tests performed in triplicate; quercetin was used as a positive control. 

### 2.7. Dermatoprotective Activity

The dermatoprotective activity of *O. basilicum* EO and aqueous extract was investigated by testing their inhibitory effect towards tyrosinase enzyme following the method described by Bouyahya and coworkers [59]. According to this method, 25 μL of EOs were added to the tyrosinase solution (100 μL, 333 units/mL in 50 mM phosphate buffer, pH 6.5) and maintained at 37 °C for 10 min. After incubation, 300 μL of L-DOPA (at 5 mM) were added, and an incubation of 30 min at 37 °C was performed. Then, the optical density (OD) was measured at 510 nm. The IC_50_ was calculated by modeling concentrations as a function of percentage concentrations. 

### 2.8. Toxicological Study

#### 2.8.1. Experimental Animals

Adult Swiss albino mice were used for acute treatment, while Wistar rats were used for 90-day toxicity studies. Animals were provided by the animal colony, Department of Pharmacology and Toxicology, Faculty of Medicine and Pharmacy, Mohamed V University (Rabat-Morocco). Animals were maintained under standard husbandry conditions (12 h light/dark cycle at room temperature of 20–22 °C). All experimental procedures were performed in accordance with the guidelines of the Ethical Committee for the care and use of laboratory animals.

#### 2.8.2. Acute Oral Toxicity Study 

The acute toxicity of *O. basilicum* EO and aqueous extract was evaluated according to the Organization of Economic Co-operation and Development (OECD) 423 standards [60]. A total of 60 mice (25–35 g) were randomly divided into six groups of 12 animals (6 males and 6 females). After an overnight fast, the EO or aqueous extract of this plant was administered orally in a single dose of 0.5, 1, 2, and 5 g/kg b.w. The treated groups had unlimited access to water and food and were controlled for 14 days.

#### 2.8.3. Chronic Toxicity Study

The chronic toxicity study was performed according to OECD test guideline 408 for 90 days with certain modifications [61]. A total of 48 male and female Wister rats (190–250 g) were randomly divided into four groups of 12 animals each (6 males and 6 females). After designing the animals into 4 groups of 6 rats each, the treated group received daily gastric gavage at increasing doses (0.5, 0.7, and 1 g/kg) of EO or aqueous extract tested. In contrast, the control group received a physiological solution (vehicle) for 90 days. During the experimental period, the body weights of all animals were measured once a week. They were observed visually for mortality rate, behavioral changes, physical appearance changes, and signs of illness.

### 2.9. Molecular Docking of EO Compounds against E. coli

#### 2.9.1. Molecular Structure Preparation

The three-dimensional structure of *E. coli* DNA gyrase B protein was obtained from the Research Collaboratory for Structural Bioinformatics (https://www.rcsb.org, accessed date on 15 July 2022, PDB ID: 4DUH) with an X-ray resolution of 1.75 Å [62]. Swiss-PdbViewer, version 4.1.0 (http://www.expasy.org/spdbv) was used for energy minimization (EM) of DNA gyrase B methyl chavicol (PubChem ID: 637563), and *trans*-anethole (PubChem ID: 637563) were considered as candidates for DNA gyrase B inhibition. The structures of SDF (structure-data file) format were obtained from the European Commission database. PubChem Pymol software was used to convert the SDF to protein data bank (PDB) format. The ligand was structurally and geometrically optimized by EM using HyperChem software, version 8.0.10.

#### 2.9.2. Pharmacokinetic, Toxicology, and Lipinski Test

The pharmacokinetic properties of OBEO main components were predicted using the SwissADME web server (http://www.swissadme.ch/. accessed date on 15 July 2022). In addition, the PreADMET web server (https://preadmet.bmdrc.kr/) was used to examine the toxicological characteristics of small molecules to predict the carcinogenicity of compounds in mouse and rat models and for the estimation of possible inhibition of the human ether-à-go-go related gene (hERG) channel in the heart. The PubChem database (https://pubchem.ncbi.nlm.nih.gov. accessed date on 15 July 2022) was used to assess the drug character of *O. basilicum* main compounds according to the theoretical method of pharmacokinetic parameters based on the rule of five (RO5) defined by Lipinski [63]. The molecular weight and octane/water partition coefficient and the number of hydrogen bond donors and acceptors in the molecule were also investigated.

#### 2.9.3. Docking Analysis

AutoDock software version 4.0 (http://autodock.scripps.edu. accessed date on 15 July 2022) was used to perform the docking analysis. The AutoDock tool predicts the conformation of the receptor-bound ligand with a predicted Gibbs free binding energy. The grid options were defined as follows; spacing (0.575 Å), X dimension (40), Y dimension (40), and Z dimension (40). The critical amino acids (AAs) are located in the active site of DNA gyrase B. These acids were selected as docking boxes [64]. Finally, BIOVIA Discovery Studio viewer version 21.1.0 was used to visualize the interaction mode between the ligand and the active site residues.

### 2.10. Statistical Analysis

All of the experiments were conducted in triplicate, and the obtained results are expressed as mean ± SD. The data were analyzed using SPSS software version 23 (IBM SPSS statistics for Windows, Version 23 Armonk, NY, USA), and comparisons between means were conducted using one-way ANOVA, followed by the Tukey test. The differences between the means were considered significant when *p* < 0.05.

## 3. Results 

### 3.1. Chemical Composition of EOs

**Figure 1:**
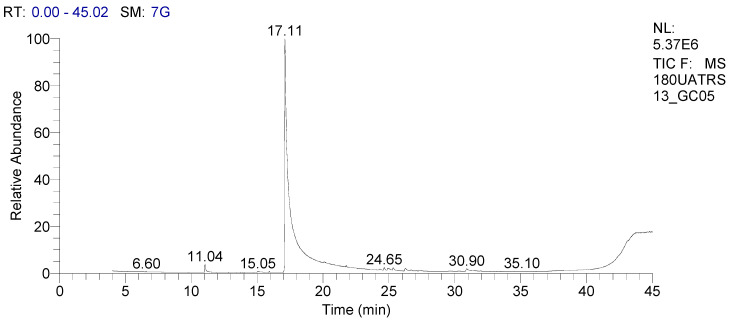
Gas chromatograph of OBEO.

The GC-MS technique determined the chemical composition of EOs. Volatile compounds were first separated by gas chromatography (Figure 1), and then each compound was identified based on its retention time (RT) and mass spectrum (MS). The chemical composition of EOs is presented in Table 1. Results show that methyl chavicol (86.40%) and *trans*-anethole (8.31%) are the main compounds of OBEO. However, several other compounds were detected at lower concentrations.

The phenolic composition of *O. basilicum* extracts was determined by some previous studies. Table 2 summarises different phenolic compounds identified in *O. basilicum* extracts. Different studies revealed the presence of numerious bioactive compounds such as chlorogenic acid, vanillic acid, epicatechin, rutin, cinnamic acid, 2,5 dihydroxybenzoic acid, 4-hydroxy benzoic acid, *p*-coumaric acid, caffeic acid, chlorogenic acid, 3,4-dihydroxy benzoic acid, gallic acid, rosmarinic acid, chicoric acid, caftaric acid, and caffeic acid. The content of each compound depended on several factors such as the type of extract, the used organ of plant, the method of extraction, as well as the method of characterization and identification.

### 3.2. Antibacterial Activity

The present work aimed to provide knowledge of the antimicrobial activity of *O. basilicum* from northern Morocco against some infectious microorganisms for human and agricultural products. The antimicrobial activity of *O. basilicum* EO and aqueous extract was first investigated using the disk diffusion assay against reference pathogenic bacteria (Gram-positive and Gram-negative) and isolated yeasts and fungi. The results are presented in Table 3. The obtained inhibitory zone values showed variations in the outcomes depending on the tested strain and the type of *O. basilicum* extract examined. Results revealed that EO exhibits potent antimicrobial, antifungal, and anti-yeast effects against all tested strains compared to the aqueous extract.

The measured inhibitory zones allowed the antimicrobial action of OBEO to be classified into strong (12 to 20 mm) and very strong (>20 mm) effects, in which *B. subtilis* ATCC 6633, S. aureus ATCC 29213, *L. monocytogenes* ATCC 13932, *C. albicans*, *T. rubrum*, and *A. niger* were the most sensible strains. On the other hand, the smallest diameter of the inhibition zone was recorded against *S. enterica* Typhimurium ATCC 700408 (a Gram-negative bacterium). Furthermore, our findings clearly indicated that all tested bacteria, as well as fungi and yeasts, were sensitive to chloramphenicol and nystatin, respectively (Table 3). Interestingly, OBEO showed the largest inhibitory zone (20.7 mm) against *B. subtilis* ATCC 6633 compared to chloramphenicol (15.7 mm).

The quantitative effect of *O. basilicum* EO and aqueous extract was evaluated using the microdilution technique and gradient plate method for bacterial, yeast, and fungal strains, respectively. The results are summarized in Table 4. Results show that OBEO has a stronger inhibitory effect than the aqueous extract against all the microorganisms tested. The lowest MIC values of OBEO were obtained against *B. subtilis* ATCC 6633 and *L. monocytogenes* ATCC 13932 (0.25 % (*v*/*v*)), and S. aureus ATCC 29213, *E. coli* ATCC 25922, and *P. mirabilis* ATCC 25,933 with a MIC value of 0.25 % (*v*/*v*). Additionally, EO showed an MIC value of 1% (*v*/*v*) against *S. enterica* Typhimurium ATCC 700408 and *C. albicans*, and an MIC equal to 2% (*v*/*v*) was achieved against fungal isolates tested (Table 4). Overall, MICs were comparable to MBCs against the tested Gram-positive bacteria indicating a bactericidal effect of OBEO. However, the double amount of MIC was the effective dose to kill Gram-negative bacteria (MBC equal to 1% against *E. coli* ATCC 25922 and *P. mirabilis* ATCC 25933, and 2% against *S. enterica* Typhimurium ATCC 700408), *C. albicans* (MBC of 2%) and fungus inoculums (MBC equal to 4% against *T. rubrum* and *A. niger*) (Table 4).

### 3.3. Antioxidant Activity

The antioxidant activity of *O. basilicum* EO and aqueous extract was determined by three complementary methods. Results are expressed in IC_50_ and summarized in Table 5. Both EO and aqueous extract exhibited interesting antioxidant properties in the three methods with some variations. The aqueous extract showed a significant inhibitory effect in the DPPH (IC_50_ = 4.12 ± 0.08 mg TEs/g), ABTS (IC_50_ = 21.00 ± 0.06 mg TEs/g), and FRAP (IC_50_ = 33.13 ± 0.17 mg TEs/g) assays compared to Eos, which showed inhibition in the DPPH, ABTS, and FRAP with IC_50_ values of 6.41 ± 0.02, 32.58 ± 0.53, and 74.66 ± 0.65 mg TEs/g, respectively. These values were lower than those recorded by the positive control (Trolox). These findings are in perfect agreement with those obtained in a similar recent study, which showed a DPPH scavenging capacity of *O. basilicum* extracts (IC_50_ = 1.29 mg/mL) greater than that of the EOs (IC_50_ = 11.23 mg/mL) [71]. This was related to the high correlation between this activity and the total phenolic content of basil extracts. Table 5 presents the antioxidant activities of *O. basilicum*.

### 3.4. Antidiabetic Activity

The in vitro antidiabetic effect of *O. basilicum* EO and aqueous extract was evaluated by testing their abilities to reduce the catalytic activities of the main enzymes involved in the catabolism of complex carbohydrates and lipids. These enzymes include α-amylase, α-glucosidase, and lipase. EOs exhibited significant inhibition of α-amylase (IC_50_ = 50.51 ± 0.32 μg/mL) and α-glucosidase (IC_50_ = 39.84 ± 1.2 μg/mL) compared to the aqueous extract, which revealed IC_50_ values of 82 ± 0.26 and 56 ± 4.21 μg/mL against α-amylase and α-glucosidase, respectively. These results are different from acarbose (used as an antidiabetic drug). Moreover, EOs and the aqueous extract of this plant also exhibited an anti-lipase inhibitory effect with IC_50_ values of 43.24 ± 0.01 and 74.28 ± 0.02, respectively, while orlistat (used as an anti-lipase drug) inhibited the activity of this enzyme with an IC_50_ of 39.84 ± 1.2 μg/mL (Table 6).

### 3.5. Anti-Inflammatory and Dermatoprotective Activities

Lipoxygenase is a crucial enzyme involved in the formation of inflammatory phenomena, and its activity contributes significantly to chronic inflammation. Consequently, its inhibition constitutes a prime therapeutic target for chronic inflammation. The results of this investigation revealed that *O. basilicum* exhibits considerable inhibitory activity against this enzyme (Table 7). Interestingly, the activity of the EO (IC_50_ = 18.28 ± 0.03 μg/mL) was higher than that of the aqueous extract (IC_50_ = 24.8 ± 0.01 μg/mL), whereas quercetin, the reference drug, showed an IC_50_ value of 4.19 ± 0.02 μg/mL. 

### 3.6. Toxicological Investigation

A safety evaluation of *O. basilicum* is required for further pharmacological investigations. Toxicity studies carried out confirm the non-toxicity of this plant, and our findings are part of this verification. In our investigation, other parameters related to toxicity and opening possible perspectives were evaluated. First, we started with an acute toxicity test by administering single doses (0.5, 1, 2, and 5 g/kg b.w.). At the end of this experiment (14th day), no mortality or sign of toxicity were recorded. Then, we conducted a chronic toxicity test to confirm this long-term harmlessness by evaluating the body weight of the animals and some of their hematological parameters for 90 days divided into four periods (D_0_, D_30_, D_60_, and D_90_); results are shown in Table 8.

At the end of each period, a significant increase in body weight compared to the first day of the experiment was observed for both administered doses (0.25 and 0.5 g/kg b.w.) compared to the control group, indicating no toxicity within the tested dose limits. At the end of the experiment, this was also confirmed by analyzing hematological parameters when no abnormalities were observed at both doses (Table 9).

### 3.7. Molecular Docking 

#### 3.7.1. Lipinski Test 

The physico-chemical characteristics of methyl chavicol and *trans*-anethole were studied based on RO5 according to the Lipinski test. According to Lipinski, the compound must have a molecular weight ≤ 500 g/mol, a LogP ≤ 5 (logarithm of the partition coefficient between *n*-octanol and water), several hydrogen bond acceptors ≤ 10, and several hydrogen bond donors ≤ 5. Based on the results displayed in Table 10, these compounds are in agreement with this test. They can be considered as oral drugs.

#### 3.7.2. Pharmacokinetic and Toxicological Properties (ADME/Tox)

The pharmacokinetic characteristics evaluated by the SwissADME web server were blood-brain barrier (BBB) permeability, gastrointestinal (GI) absorption, P-gp (P glycoprotein) substrate, cytochrome P-450 inhibition, and skin permeation coefficient (kp). Possible inhibition of the human cardiac potassium channel hERG and carcinogenicity of the compounds in animal models (mouse and rat) were considered toxicological properties. Methyl chavicol and *trans*-anethole were predicted to have lower toxicity effects. ADMET data from this study are given in Table 11.

#### 3.7.3. Molecular Docking

**Figure 2:**
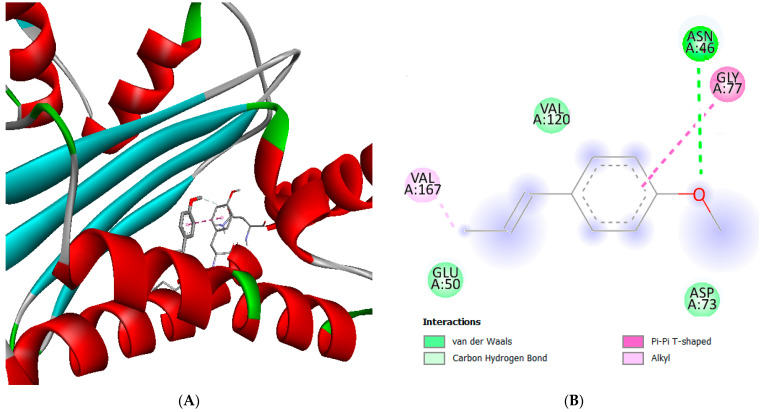
2D diagram (**A**) and 3D representation (**B**) of the docking pose of the inhibitor *trans*-anethole in the active site of *E. coli* DNA gyrase B (PDB ID: 4DUH) with an RMSD of 0.575 Å.

**Figure 3:**
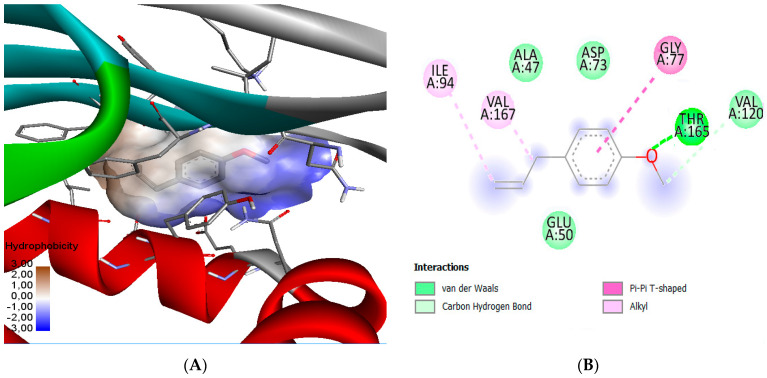
2D diagram (**A**) and 3D representation (**B**) of the docking pose of the inhibitor methyl chavicol in the active site of *E. coli* DNA gyrase B (PDB ID: 4DUH) with an RMSD of 0.575 Å.

The molecular docking approach was performed to assess the binding affinity of the compounds to the active site of DNA gyrase B. Table 12 lists the binding energies between DNA gyrase B and the compounds tested in this study. Figure 2 and Figure 3 show the images of 3D and 2D ligand-AA interactions for both studied compounds. Comparison of the residues included in the active site with that interacting with these compounds reveals AAs in common, which confirms the hypothesis that these compounds exhibit the most inhibitory effect of *E. coli* [72]. The *trans*-anethole is surrounded by Van der Waals bonds from the AAs Val120 and Glu50 and an alkyl bond with Val 71; Gly77 interacts with the benzene ring of the compound through a pi-pi cationic bond. Asp73 establishes a carbon-hydrogen bond with the compound’s electron-accepting carbon; the only hydrogen bond is that of Asn46 with the oxygen atom. In addition, methyl chavicol shows significant interactions with *trans*-anethole. This compound is surrounded by the hydrophobic chains of the AAs Ala47, Glu50, Asp73, and Val120, and alkyl bonds with Ile94 and Val167. Similar to *trans*-anethole, the benzene ring of chavicol interacts with the Gly77 residue via a pi-pi cationic bond. The compound is extended into the active site outwards by completing a hydrogen bonding interaction with the critical AAs Thr165.

## 4. Discussion

The chemical composition of OBEO is different from those reported in the literature. Indeed, a study by Al-Maskri et al. [73] showed a variation in OBEO chemical composition [73]. This difference could be attributed to several factors, including geographical origin, climate, and phenological stage. In addition, the chemical analysis showed the richness of OBEO in phenolic compounds, which justifies its biological properties. This richness has also been confirmed in *O. basilicum* extracts by the high-performance liquid chromatography (HPLC) method in previous studies. According to the study, the ethanolic extract of *O. basilicum* presented a variety of major molecules, namely rutin (476.28 μg/g), epicatechin (225.01 μg/g) [69] rosmarinic acid (360.40 ± 1.28 μg/g), vanillic acid (36.30 ± 0.25 μg/g) [74], and benzoic acid (50.86 mg/g) [75]. In addition, *O. basilicum* phenolic extract had catechin (6300.736 μg/mL) as the main compound [76]. Antimicrobial investigations showed important results. Most of the comparative articles on aromatic and medicinal plants agree that the sensitivity of Gram-positive bacteria is higher than that of Gram-negative with respect to the inhibitory action of EOs [69,77]. However, all Gram-negative and Gram-positive bacteria studied were sensible to OBEO with varying degrees of response. In this regard, the present study showed a potent antibacterial effect of OBEO against the tested Gram-positive bacteria by both the disk diffusion (inhibitory zones between 17.2 and 20.7 mm) and the broth microdilution techniques (MIC values between 0.25 to 0.5% *v*/*v*). These results are in agreement with those obtained in previous research. Hussain et al. [77] reported that *O. basilicum* aerial part EO from Pakistan exhibits similar antibacterial action, with inhibitory zones ranging from 26.1 ± 1.1 mm to 24.0 ± 1.0 mm and MIC values fluctuating between 0.8 ± 00 and 0.9 ± 00 mg/mL against *B. subtilis* and *S. aureus*, respectively. Shirazi et al. [78] studied the antibacterial activity of OBEO and showed complete growth inhibition of *S. aureus and B. subtilis* with MIC values of 67 ± 8 and 75 ± 7 μg/mL, respectively. In addition, the study of the antibacterial properties of EOs and methanol extracts of *O. basilicum* from Bangladesh showed considerable activity against *L. monocytogenes* [79].

Recently, a study was conducted on OBEO against streptomycin and colistin-resistant *E. coli* isolates from the turkey’s upper thigh and skin. Results showed inhibitory zones of 12.25 and 20.66 mm against *E. coli* isolated from the turkey’s upper thigh and skin, respectively [80]. Additionally, all *E. coli* strains were killed at a MBC of 1/2000 (*v*/*v*) [80]. In the same context, an Indian OBEO exhibited bactericidal action against Gram-negative bacteria obtained from India’s National Collection of Industrial Microorganisms (NCIM) [23]. Indeed, the studied strains of *P. mirabilis* (NCIM 2241), *S. enterica* Typhimurium (NCIM 2501), and *E. coli* (NCIM 2574) were found to be moderately sensible to OBEO, with MBC values of 0.781 ± 0.382, 1.562 ± 0.765, and 1.666 ± 0.645 mg/mL, respectively [23].

The advancement in scientific therapies and applied sciences has led to the development of particles through nanotechnology. In this context, Malapermal et al. [81] performed an approach to enhance the antimicrobial activity of *O. basilicum* leaf aqueous extracts using silver nanoparticles (AgNps). Using AgNps bio-derived from *O. basilicum*, a high antibacterial effect against *E. coli* was observed, while a weak activity was noticed against *P. aeruginosa* [81]. Nonetheless, the AgNps synthesized from *O. basilicum* leaf aqueous extracts were not active against *Salmonella* species [81]. Compared to our anti-yeast findings against *C. albicans*, Rezzoug et al. [69] demonstrated a higher MIC of *O. basilicum* ethanolic extract (64 μg/mL) to that of EOs (32 μg/mL) against the same yeast species. Likewise, *C. albicans* was slightly resistant to *O. basilicum* methanolic extract [82].

Currently, it is well established that the fluctuation in antimicrobial activity between different research works is mainly due to variability in the chemical composition. The main chemotypes of OBEO are conditioned by environmental factors, cultivation, soil chemical components, analytical methods, storage and processing conditions, collection period, and other circumstances [73,77]. In addition, the extraction method used can affect the results. Our outcomes are presented in Table 2 and Table 3. Interestingly, the relevant findings of Rezzoug et al. [69] indicated such fluctuations using two extraction methods for *O. basilicum*. They explained the difference in the results by the yield and percentage of bioactive content obtained by each extraction technique. Consequently, OBEOs exhibit significant antimicrobial activity compared to other by-products, particularly aqueous, methanolic, and ethanolic extracts. Furthermore, the growth of *Trichophyton mentagrophytes* (a keratinophilic fungus) and *A. niger*, as well as the spoilage agent of fruits and vegetables were affected by OBEO. The inhibitory zones varied from 15.6 to 17.3 mm and the MIC value was equal to 2% (*v*/*v*) against both tested species. Our results are consistent with those of previous surveys [73,83]. In contrast, Ibrahim and Abd El-Salam [84] noticed a better growth inhibitory activity of OBEO at concentrations of 0.5 and 1 μL/mL against *T. rubrum* compared to our findings. Similarly, basil EO collected from Oman presented a MIC of 50 μg/mL for *A. niger* [73].

*O. basilicum* belongs to the Lamiaceae family, exhibiting various significant activities due to its high content of bioactive secondary metabolites such as phenolic and flavonoid constituents. Several substances were reported as the main phytochemicals of OBEO, in particular linalool (Oman and Algeria) [69,80,85], methyl eugenol (India) [23], methyl chavicol (Pakistan) [79], and citronellal (Spain) [86]. In the present study, methyl chavicol and *trans*-anethole were the most abundant components in OBEOs obtained in Taounate, northern Morocco. Key phytochemicals show promising antimicrobial properties compared to previous investigations of nearby regions [69,80,86]. Thus, methyl chavicol is mainly a natural and safe alternative component suitable for human therapy and reducing environmental pathogens. Recently, researchers have become more aware of the microbial growth repression mechanisms of the isolated pure bioactive phytochemicals. The investigation conducted by Miao and coworkers [87] on metabolomic analysis of *C. albicans* treated with basil EOs showed that the abundance of 34 from 140 intracellular metabolites of the examined microorganism changed significantly after this oil treatment, including those involved in central carbon, AA, polyamine, and lipid metabolism. Notably, the investigation also shed light on the role of the minor molecules in a synergistic interaction with other chemotypes [87].

The results of the antidiabetic effects corroborate those observed in several investigations evaluating the in vitro antidiabetic activity of *O. basilicum*, particularly by targeting carbohydrate-hydrolyzing enzymes, namely α-amylase and α-glucosidase [88,89,90,91]. Another study by Ezeani et al. [92] assessed the antidiabetic activity in vitro and in vivo. The authors administered increasing doses of the extracts of this plant for 28 days to alloxan-induced diabetic rats. Consequently, several positive results were noted, namely a reduction in fasting blood glucose, an increase in hepatic glycogen content, and an improvement in glucose tolerance. Other in vivo research has also investigated how this species can reduce high blood glucose levels. Diabetes was induced in laboratory animals either by streptozotocin (STZ) or by alloxan. *O. basilicum* extracts and EOs revealed several mechanisms of diabetes management, such as modulation of biochemical, morphometric, and histological disorders of colonic tissue, increased activities of plasma ALT, lysozyme, and total protein, decreased levels of triglycerides, plasma glucose, and hepatic glycogen [93,94,95]. From the phytochemical section of our work and the results of Mousavi et al. [96], basil showed richness in active polyphenolic components such as rosmarinic acid, azulene, luteolin, kaempferol, genistein, caffeic acid, diosmetin, 3,4-dimethoxycinnamic acid, and apigenin, which are known to control blood glucose in diabetic animals.

In fact, it is quite evident that the anti-diabetic potential of basil is strongly attributed to its phytochemical composition. As mentioned above, HPLC methods of *O. basilicum* extracts have recently confirmed the presence of many major compounds (rutin, epicatechin, catechin, vanillic acid, rosmarinic acid, and benzoic acid) with remarkable antidiabetic properties. Indeed, in nicotinamide (NA)/STZ-induced diabetic rats, administration of a daily oral dose of epicatechin (10 mg/kg) for 4 weeks significantly normalized glucose tolerance, insulin resistin, glucose transporter type 4 (GLUT4) mRNA expression, and serum insulin level [97]. Another compound of the flavonoid family called catechin, has also shown a hypoglycemic effect in mice and humans by suppressing the increase in postprandial glucose [98]. This effect was later confirmed with rutin using the oral glucose tolerance test (OGTT), after administration of a dose of 10 mg/kg for 28 days to alloxan-induced diabetic mice [99]. Moreover, in NA/STZ induced diabetic rats, a 6 week oral treatment with vanillic acid at increasing doses 25, 50, and 100 mg/kg significantly lowered blood glucose, improved lipid profile, and increased liver enzymes [100]. Furthermore, the anti-diabetic effect of other main compounds has also been confirmed in in vitro studies. Benzoic acid [101] and rosmarinic acid [102] showed strong inhibitory effects on α-glucosidase enzyme activity.

Our findings on anti-inflammatory properties are consistent with those reported by Benedec et al. [103]. The anti-inflammatory action of *O. basilicum* extract was lower than that of the reference drug, diclofenac [103]. In addition, we share the same results with another previous study, which showed lipoxygenase enzyme inhibition of 98.2% at a concentration of 8 mg/mL OBEO [104]. Furthermore, the extract of this plant inhibits the production of specific inflammatory mediators, namely iNOS (inducible nitric oxide synthase) and NO (nitric oxide) [105]. The same anti-inflammatory action was observed in vitro with *O. basilicum* leaf ethanolic extract on lipopolysaccharide (LPS)-stimulated RAW 264.7 cells by inhibiting NO production [106]. Moreover, specific pro-inflammatory cytokines (IL-2, IL-β, and TNF-α) and inflammatory mediators (iNOS and NO) were inhibited by the action of *O. basilicum* methanolic extract [107]. The observed effects of basil could be attributed to its compounds and those that are in the minority or to the synergy between them. Indeed, according to Rodrigues et al. [108], OBEO and its major molecule, estragole, effectively manage inflammation, whether acute or chronic.

Indeed, according to very recent studies, this activity is related to the anti-inflammatory potential of *O. basilicum* bioactive compounds. In fact, Tjahjono et al. [109] showed that administration of an oral dose of benzoic acid (500 mg/60 kg b.w.) to LPS-treated rats reduced levels of IL-1β (2.32 ± 0.28 × 10^3^ pg/mL) and tumor necrosis factor-α (TNF-α) (5.70 ± 1.04 × 10^3^ pg/mL), as well as the severity of lung damage and white blood cell concentration. Similarly, using LPS-stimulated immortalized mouse Kupffer cells (ImKCs), rutin inhibited NO synthesis via down-regulation of iNOS expression [110]. The authors attributed this effect to inhibition of nuclear factor (NF)-κB activation and regulation of expression of certain pro-inflammatory cytokines (IL-1β and IL-6). Using the same experimental model, Kim et al. [111] evaluated the anti-inflammatory effect of catechin-7,4′-O-digallate on the reduction of NO production. The results showed an inhibition of the production of this free radical via the downregulation of iNOS expression. In addition, an epicatechin dimer reduced carrageenan-induced paw edema in female Wistar rats [112]. This reduction in the inflammatory response was similarly observed with rosmarinic acid against non-alcoholic steatohepatitis (NASH), which was induced by a methionine-choline-deficient (MCD) diet in C57/BL6 mice [113]. Indeed, rosmarinic acid reduced the expressions of IL-6, TNF-α, and P53 via activation of the sirtuin 1 (SIRT1)/nuclear factor (NF)-κB pathway. Moreover, a significant attenuation of ocular inflammation was recorded with the formulation of vanillic acid in spanlastic nanovesicles [114].

On the other hand, certain enzymes are involved in the skin aging process. In fact, tyrosinase is responsible for skin deterioration by decreasing the maintenance property of skin cells. The inhibition of this enzyme, therefore, constitutes a dermato-protective approach. For this reason, we chose this enzyme as a target in our study. As we observed with the previous enzyme (5-lipoxygenase), the EO of our plant displayed significant values (IC_50_ = 68.58 ± 0.03 μg/mL) compared to the aqueous extract (IC_50_ = 118.37 ± 0.05 μg/mL) in comparison with the standard reference (IC_50_ = 25.08 ± 0.12 μg/mL). This suggests that this plant can protect the skin against burns, wounds, and lesions, among others, and treat skin disorders such as melanoma and hyperpigmentation [110]. Several recent investigations have supported our findings. In this line, Avetisyan and co-workers recorded tyrosinase inhibitory activity of basil EOs from different cultivars, namely *O. basilicum* var. *thyrsiflora* (20.1 ± 1.4%) and *O. basilicum* var. *purpureum* (11.5 ± 0.3%) vs. positive control, arbutin (81.5 ± 2.6%) [111]. Similarly, more recent studies have indicated a moderate inhibition of tyrosinase obtained by extracts and EOs compared to positive controls [74,112]. However, studies on EOs from *O. basilicum* leafy stems showed considerable activity in inhibiting this enzyme (79.72 ± 8.08 mg KAE/g) [90]. In the same study, the major molecule, methyl chavicol (51.9%), also identified in our case, was suggested by in silico studies as an inhibitor of tyrosinase activity. This tyrosinase inhibitory potential is probably attributed to the antioxidant effect. This enzyme is responsible for the oxidation of phenols, and its inhibition can reduce melanin secretion, which explains its use in cosmetics.

The safety of *O. basilicum* is supported by its hepatic and renal protective effects often attributed to its bioactive molecules. In this respect, Soliman et al. [94] evaluated this protective effect against paracetamol-induced hepatorenal toxicity in rats by measuring biochemical parameters (transaminases, bilirubin, albumin, creatinine, urea, etc.) as well as hepatic and renal histopathology for 30 days. The administration of daily doses of *O. basilicum* ethanolic extract (200 and 400 mg/kg b.w.) restored the values of liver and kidney parameters altered by paracetamol toxicity and reduced the histopathological liver and kidney lesions caused by this toxicity. Likewise, in carbon tetrachloride (CCl_4_)-induced liver toxicity in rats, a rosmarinic acid-rich extract from *O. basilicum* did not cause any signs of toxicity or mortality [113]. In contrast, at a dose of 200 mg/kg b.w., it significantly lowered liver enzyme activities. From these data, it can be concluded that *O. basilicum* is harmless. However, other investigations are necessary to complete its toxicological profile by evaluating the toxicity of higher doses on the target organs (liver and kidneys) and the intestines since they constitute the site of toxin absorption; and this process as a function of time over different periods, following the example of the experimental protocol adopted in the toxicological study conducted by El Omari et al. [114].

## 5. Conclusions and Perspectives

The present work was carried out to study the biological properties of *O. basilicum*. The results revealed that the plant contains volatile compounds with a significant diversity with the presence of two major compounds (methyl chavicol and *trans*-anethole) and a predominance of methyl chavicol constituting 86%. Analysis of the biological activities highlighted the antibacterial, antidiabetic, anti-inflammatory, and dermatoprotective effects of EOs and aqueous extracts of this plant. In addition, toxicological investigations have verified the harmlessness of this plant widely used in traditional Moroccan medicine. Considering the important antibacterial effects of EO, an in-silico study was carried out to characterize the molecular interactions between the two primary compounds identified in the EO and the possible antibacterial targets. Therefore, significant reactivity of both compounds was noted regarding specific antibacterial targets. Despite these promising results demonstrated in this work, further investigations seem essential to validate the safety of this medicinal species as a function of dose and time. Experimental studies concerning the mechanisms of action of the two major compounds would be essential to validate their pharmacological properties.

## Figures and Tables

**Table 1:** Chemical composition of OBEO.

Compounds	RT (min)	%
Eucalyptol	11.05	0.74
α-campholene aldehyde	15.06	0.23
Cyclopentasiloxane, decamethyl-	15.92	0.05
Methyl chavicol	17.12	86.40
*Trans*-anethol	20.07	8.31
*Cis*-anethol	21.76	1.64
Calarene	24.65	0.18
α-Longipinene	24.93	0.35
Azulene	25.33	0.31
Aristolene	26.25	0.33
Cedrene	26.67	0.09
Aristolene	27.35	0.11
Azulene	30.91	0.48

**Table 2:** Chemical composition of *O. basilicum* extracts.

Phytochemical Compound	Reference
Rosmarinic acid, Chicoric acid, Caftaric acid, Caffeic acid	[65]
Caftaric acid, gentisic acic, caffeic acid, chlorogenic acid, *p*-coumaric acid, ferulic acid, isoquercitrin, rosmarinic acid, rutin, quercitrin, quercetin, luteolin	[66]
Caffeic acid, caftaric acid, chicoric acid, gentisic acid, rosmarinic acid	[67]
Caftaric acid, caffeic acid, ferulic acid, chicoric acid, rosmarinic acid	[68]
Chlorogenic acid, vanillic acid, epicatechin, rutin, cinnamic acid, 2,5 dihydroxybenzoic acid, 4-hydroxy benzoic acid, *p*-coumaric acid, caffeic acid, chlorogenic acid, 3,4-dihydroxy benzoic acid, gallic acid	[69]
Caffeic, caftaric, chicoric, gentisic, *p*-coumaric, and rosmarinic acids	[70]

**Table 3:** Diameters of the inhibition zones (mm) of *O. basilicum* EO and aqueous extract.

Microorganisms	***Ocimum basilicum***		Controls	
	OBEO	Aqueous Extract	Chloramphenicol	Nystatin
*Escherichia coli* ATCC 25922	16.5 ± 0.20	12.6 ± 0.20	21.9 ± 0.20	NT
*Proteus mirabilis* ATCC 25933	15.4 ± 0.25	11.6 ± 0.15	22.2 ± 0.15	NT
*Salmonella enterica* Typhimurium ATCC700408	12.1 ± 0.05	8.0 ± 0.50	13.5 ± 0.15	NT
*Bacillus subtilis* ATCC 6633	20.4 ± 0.30	14.1 ± 0.05	15.5 ± 0.15	NT
*Staphylococcus aureus* ATCC 29213	17.3 ± 0.17	13.3 ± 0.15	25.0 ± 0.25	NT
*Listeria monocytogenes* ATCC 13932	19.0 ± 0.25	13.1 ± 0.10	27.9 ± 0.15	NT
*Candida albicans*	17.3 ± 0.15	12.3 ± 0.20	NT	29.7 ± 0.20
*Trichophyton rubrum*	15.2 ± 0.36	10.4 ± 0.20	NT	24.9 ± 0.26
*Aspergillus niger*	17.1 ± 0.26	11.0 ± 0.20	NT	26.4 ± 0.10

NT: not tested.

**Table 4:** MIC and MBC of *O. basilicum* EO and aqueous extract in percentage (*v*/*v*).

Microorganisms	***O. basilicum* in % (*v*/*v*)**				Controls (µg/mL)	
	Essential Oil		Aqueous Extract		Chloramphenicol	Nystatin
	MIC	MBC	MIC	MBC		
*Escherichia coli* ATCC 25922	0.5	1	1	2	4	NT
*Proteus mirabilis* ATCC 25933	0.5	1	1	2	4	NT
*Salmonella enterica* Typhimurium ATCC 700408	1	2	4	>4	64	NT
*Bacillus subtilis* ATCC 6633	0.25	0.25	1	1	32	NT
*Staphylococcus aureus* ATCC 29213	0.5	0.5	1	1	4	NT
*Listeria monocytogenes* ATCC 13932	0.25	0.25	1	1	2	NT
*Candida albicans*	1	>4	2	NT	NT	4
*Trichophyton rubrum*	2	>4	4	NT	NT	16
*Aspergillus niger*	2	>4	4	NT	NT	16

NT: Not tested.

**Table 5:** IC_50_ values (mg TEs/g) of the antioxidant activities of *O. basilicum*.

*Ocimum basilicum*	DPPH	ABTS	FRAP
Essential oils	6.41 ± 0.02 ^a^	32.58 ± 0.53 ^a^	74.66 ± 0.65 ^a^
Aqueous extract	4.12 ± 0.08 ^b^	21.00 ± 0.06 ^b^	33.13 ± 0.17 ^b^
Trolox	2.23 ± 0.02 ^c^	9.33 ± 0.07 ^c^	8.52 ± 0.17 ^c^

Results were expressed as the mean of triplicates ± SD. Different superscript letters (a, b, and c) in the same column indicate a significant difference (*p* < 0.05).

**Table 6:** Activity of *O. basilicum* to inhibit digestive enzymes (α-glucosidase, α-amylase, and lipase).

IC_50_ (μg/mL)	α-Amylase	α-Glucosidase	Lipase
Essential oil	50.51 ± 0.32 ^a^	39.84 ± 1.2 ^a^	43.24 ± 0.01 ^a^
Aqueous extract	82 ± 0.26 ^b^	56 ± 4.21 ^b^	74.28 ± 0.02 ^b^
Acarbose	28.24 ± 0.06 ^c^	19 ± 1.12 ^c^	
Orlistat			18.27 ± 0.03 ^c^

Different letters (a, b, and c) indicate significant differences (*p* < 0.05; n = 3).

**Table 7:** In vitro anti-inflammatory and dermatoprotective activities of *O. basilicum*.

Assays	*Ocimum basilicum* IC_50_ (μg/mL)		Control
	Essential Oil	Aqueous Extract	Quercetin
5-Lipoxygenase	18.28 ± 0.03 ^a^	24.8 ± 0.01 ^b^	4.19 ± 0.0 2 ^c^
Tyrosinase	68.58 ± 0.03 ^a^	118.37 ± 0.05 ^b^	25.08 ± 0.12 ^c^

Different letters (a, b, and c) indicate significant differences (*p* < 0.05; n = 3).

**Table 8:** Body weight changes in rats treated with *O. basilicum*.

Days	Control	0.25 g/kg	0.5 g/kg
D_0_	217.97 ± 0.03 ^a^	209.46 ± 0.02 ^b^	209.26 ± 09.04 ^b^
D_30_	233.93 ± 0.01 ^a^	215.37 ± 0.01 ^b^	225.31 ± 02.14 ^c^
D_60_	244.62 ± 0.02 ^a^	226.17 ± 0.02 ^b^	254.93 ± 18.03 ^c^
D_90_	262.21 ± 0.04 ^a^	237.58 ± 0.01 ^b^	275.42 ± 39.02 ^c^

Different letters (a, b, and c) indicate significant differences (*p* < 0.05; n = 3).

**Table 9:** Hematological parameters of male rats after 90 days of treatment with *O. basilicum*.

	Control	0.25 g/kg	0.5 g/kg
Red blood cells (10^6^ μL^−1^)	8.2 ± 0.1 ^a^	8.4 ± 0.2 ^b^	8.5 ± 0.1 ^b^
White blood cells (10^3^ μL^−1^)	12.4 ± 0.2 ^a^	12.8 ± 0.1 ^b^	12.9 ± 0.3 ^b^
Hemoglobin (g/dL)	11.8 ± 0.2 ^a^	12.2 ± 0.4 ^b^	12.3 ± 0.5 ^b^
Hematocrit (vol %)	42.2 ± 0.8 ^a^	45.5 ± 1.4 ^b^	47.8 ± 1.3 ^c^
Platelets (×10^4^ L^−1^)	82.8 ± 0.2 ^a^	84.6 ± 0.3 ^b^	85.9 ± 0.1 ^c^
Lymphocytes (%)	78.31 ± 0.3 ^a^	79.45 ± 0.5 ^b^	79.68 ± 0.3 ^b^
Neutrophils (%)	15.5 ± 0.3 ^a^	15.6 ± 0.8 ^a^	15.9 ± 0.5 ^a^

Different letters (a, b, and c) indicate significant differences (*p* < 0.05; n = 3).

**Table 10:** Properties of Lipinski’s RO5 associated with methyl chavicol and *trans*-anethole.

Ligand Name	Molecular Weight (g/mol)	XLogP3 Count	Hydrogen Bond Donor	Hydrogen Bond Acceptor Count	Agreement with RO5
Methyl chavicol	148. 2	3.4	0	1	Yes
*Trans*-anethole	148.2	3.3	0	1	Yes

**Table 11:** Predicted pharmacokinetic and toxicological properties of methyl chavicol and *trans*-anethole.

Ligand	GI Abs	Blood-Brain Barrier Permeant	P-Glycoprote in Substrate	CYP1A2 Inhibitor	CYP2C19 Inhibitor	CYP2C9 Inhibitor	CYP2D6 Inhibitor	CYP3A4 Inhibitor	Log Kp	Carcino_Mous	Carcino_ Rat	hERC Inhibition
Methyl chavicol	High	Yes	No	Yes	No	No	No	No	−4.73	Positive	Positive	Medium risk
*Trans*-anethole	High	Yes	No	Yes	No	No	No	No	−4.87	Positive	Positive	Medium risk

**Table 12:** Details of binding energies between DNA gyrase B and methyl chavicol and *trans*-anethole.

Ligand ID	Ligand Name	Final Intermolecular Energy (kcal.mol)	Final Total Internal Energy (kcal.mol)	Torsional FreeEnergy (kcal.mol)	Unbound System Energy (kcal.mol)	Estimated Free Energy of Binding (kcal/mol)
8815	Methyl chavicol	−5.28	−0.19	0.86	−0.16	−5.21
637563	*Trans*-anethole	−6.08	−0.18	0.89	−0.15	−5.22

## Data Availability

All generated data have been included in the manuscript.

## Sample Availability

Samples of the compounds are available from the authors.

## Abbreviations

AA:	Amino Acids
ABTS:	2,2-Azinobis(3-ethyl-Benzothiazoline-6-Sulfonic acid
ATCC:	American Type Culture Collection
BBB:	Blood-Brain Barrier
CCl_4_:	Carbon Tetrachloride
DMSO:	Dimethyl Sulfoxide
DNA:	Deoxyribonucleic Acid
DPPH:	2,2- Diphenyl-1-Picrylhydrazyl
EM:	Energy Minimization
EO:	Essential Oil
FRAP:	Ferric Reducing Antioxidant Power
GC/MS:	Gas Chromatography/Mass Spectrometry
GI:	Gastrointestinal
GLUT4:	Glucose Transporter Type 4
hERG:	Human Ether-À-Go-Go Related Gene
HPLC:	High-Performance Liquid Chromatography
IC_50_:	Inhibitory Concentration 50%
IL-1:	Interleukin-1
ImKCs:	Immortalized mouse Kupffer Cells
iNOS:	inducible Nitric Oxide Synthase
LPS:	Lipopolysaccharide
MBC:	Minimum Bactericidal Concentration
MCD:	Methionine-Choline-Deficient
NCIM:	National Collection of Industrial Microorganisms
MDR:	Multidrug-Resistant
MHA:	Mueller-Hinton Agar
MHB:	Mueller-Hinton Broth
MIC:	Minimum Inhibitory Concentration
mRNA:	messenger Ribonucleic Acid
MS:	Mass Spectrum
NA:	Nicotinamide
NASH:	Non-Alcoholic Steatohepatitis
NF-κB:	Nuclear Factor-κB
NT:	Not Tested
OBEO:	*Ocimum Basilicum* Essential Oil
OD:	Optical Density
OECD:	Organization of Economic Co-operation and Development
OGTT:	Oral Glucose Tolerance Test
NO:	Nitric Oxide
PDB:	Protein Data Bank
P-gp:	P-glycoprotein
RO5:	Rule Of Five
RT:	Retention Time
SA:	Sabouraud’s Agar
SD:	Standard Deviation
SDF:	Structure-Data File
SIRT1:	Sirtuin 1
TNF-α:	Tumor Necrosis Factor-α
TSA:	Tryptone Soy Agar
WHO:	World Health Organization
5-LOX:	5-Lipoxygenase
